# Age-dependent effects of surgical approach in T3b differentiated thyroid carcinoma: a population-based analysis using machine learning

**DOI:** 10.1530/ERC-25-0417

**Published:** 2025-12-17

**Authors:** Yifan Liu, Zhuo Chen, Botao Sun, Hui Ouyang

**Affiliations:** ^1^ Department of General Surgery, Xiangya Hospital, Central South University, Changsha, Hunan Province, China; ^2^ National Clinical Research Center for Geriatric Disorders, Xiangya Hospital, Central South University, Changsha, Hunan Province, China; ^3^ Department of Gynecology, Xiangya Hospital, Central South University, Changsha, Hunan Province, China

**Keywords:** differentiated thyroid carcinoma, SEER, gross strap muscle invasion (T3b), machine learning, interaction analysis

## Abstract

Current guidelines recommend total thyroidectomy for all T3b differentiated thyroid carcinoma (DTC) with gross strap muscle invasion, yet evidence supporting this universal approach remains limited and conflicting. We analyzed 6,920 T3b DTC patients from the SEER database (2004–2022) who underwent lobectomy (*n* = 282) or total thyroidectomy (*n* = 6,638). Overall survival (OS) served as the primary outcome. Random survival forest (RSF) with SHapley Additive exPlanations (SHAP) analysis identified interactions between surgical approach and clinical variables. Cox regression with restricted cubic splines examined age-dependent surgical effects on OS. Neither RSF nor Cox regression (HR 0.96, 95% CI: 0.68–1.37, *P* = 0.84) showed a significant OS difference between surgical approaches. SHAP interaction analysis revealed significant age-dependent heterogeneity. Restricted cubic splines identified age 45 as the threshold where the surgical benefit converged. In patients <45 years, total thyroidectomy demonstrated significantly superior survival outcomes compared with lobectomy (adjusted HR 3.26, 95% CI: 1.30–8.22, *P* = 0.012; 10-year survival 95.5 vs 98.4%). Conversely, no difference existed in patients ≥45 years (HR 0.85, 95% CI: 0.58–1.24, *P* = 0.40). The multiplicative interaction was 0.26 (95% CI: 0.10–0.70, *P* = 0.008), confirming a negative age–surgery interaction. Although this study demonstrates that surgical approach shows no significant survival difference in T3b DTC, the negative interaction effect between surgical approach and age indicates that the benefit of total thyroidectomy progressively diminishes with advancing age, providing a survival advantage only in patients younger than 45 years. These findings challenge universal total thyroidectomy recommendations and support age-stratified surgical decision-making for T3b DTC.

## Introduction

The management of differentiated thyroid carcinoma (DTC) is shifting from uniformly aggressive treatment toward risk-stratified approaches (1). This evolution is particularly relevant for T3b disease – tumors with gross extrathyroidal extension limited to strap muscles – which represents an intermediate-risk category between intrathyroidal tumors and extensively invasive disease (1, 2). Despite current American Thyroid Association guidelines mandating total thyroidectomy for all gross extrathyroidal extension (1), emerging evidence questions this universal approach.

Several meta-analyses have shown that T3b disease does not significantly impact recurrence or mortality in the absence of concurrent high-risk features, such as lymph node metastasis or large tumor size (3, 4). Several Asian centers subsequently reported comparable outcomes between lobectomy and total thyroidectomy for selected T3b patients (5, 6). Most recently, tumor size within the T3b category emerged as potentially more prognostically important than strap muscle invasion itself (7). These findings suggest that selected T3b patients might avoid total thyroidectomy and its associated risks.

However, existing evidence remains fundamentally limited. Current studies (5, 6) are predominantly single-center analyses with insufficient outcome events – typically reporting fewer deaths despite extended follow-up. Selection bias and marked outcome heterogeneity across centers indicate that surgical effects likely depend on undefined patient characteristics. In addition, the prognosis heterogeneity of T3b patients across age (8, 9), tumor size (7, 9, 10), and nodal status (4, 5) might suggest the presence of effect modifiers – clinical factors that interact with surgical approach to influence outcomes in T3b patients.

Traditional statistical methods are inadequate for detecting the complex interactions that likely influence surgical outcomes in T3b disease. Random survival forests (RSFs) with SHapley Additive exPlanations (SHAP) analysis offer a powerful alternative, capable of automatically detecting non-linear relationships and high-order interactions without pre-specification (11, 12). These machine learning approaches have successfully assessed variable effects and identified clinically important interactions.

Therefore, this study leveraged the Surveillance, Epidemiology, and End Results (SEER) database to comprehensively evaluate surgical approach effects in T3b DTC. Using RSF modeling with SHAP analysis, we aimed to: i) assess the effect of surgical approach; ii) identify important clinical variables that interact with surgical approach to influence overall survival (OS); and iii) validate findings using traditional Cox regression. This systematic exploration of treatment effect heterogeneity seeks to establish evidence-based criteria for personalized surgical management in T3b DTC.

## Methods

### Patients and data collection

Data for patients diagnosed with papillary thyroid carcinoma (ICD-O-3 codes: 8050, 8260, 8340–8344, 8350, 8450–8460), follicular thyroid carcinoma (ICD-O-3 codes: 8330–8332, 8335, 8339), and Hürthle cell thyroid carcinoma (HTC; ICD-O-3 codes: 8290) were extracted from the SEER database (Incidence – SEER Research Data, 17 Registries, Nov 2024 Sub (2000–2022)). We included DTC patients diagnosed between 2004 and 2022 due to the availability of tumor extension information. Inclusion criteria included the diagnosis of DTC as the first primary cancer, confirmation of diagnosis through histological or cytological pathology, and the absence of undifferentiated or anaplastic features in histological grading. Patients were excluded if they presented with age ≤18, extrathyroidal extension beyond strap muscle invasion (T3b), unknown N stage, distant metastases (M1), absence of surgical resection, missing demographic or clinicopathological data, unavailable treatment information, unknown survival status, or follow-up duration <1 month. Through this selection process, 6,920 eligible patients were identified for final analysis (Supplementary Fig. 1 (see section on Supplementary materials given at the end of the article)). Given the publicly available nature of the SEER database, this study did not require review by the Institutional Review Board of Central South University.

### Variable collection

Comprehensive variables were collected across three domains. Demographic characteristics encompassed age at diagnosis, sex, race, marital status, household income, and county type (urban/rural). Clinicopathological features included year of diagnosis, histological grade, tumor size, multifocality status, and N stage (per 8th edition AJCC TNM classification). Treatment variables comprised surgery type (lobectomy versus total thyroidectomy) and radioactive iodine (RAI) therapy administration. OS served as the primary outcome, defined as time from diagnosis to death from any cause or last follow-up.

### Statistical analysis

An RSF model was developed to address limitations of traditional Cox regression in handling multicollinearity and high-dimensional data (11, 12). The model comprised 1,000 trees constructed using 0.632 subsampling without replacement. Node splitting employed the log-rank rule with default random splits for the node-specific variable set. Optimal terminal node size was determined through grid search methodology minimizing out-of-bag error.

SHAP methodology (11, 13) was employed to quantify feature contributions through three components. Total SHAP values represent the comprehensive contribution of a feature to survival prediction across all variable combinations, incorporating complex interactions. Main SHAP values isolate the direct, independent contribution when other variables are maintained at baseline, representing the feature’s additive effect. Divergence between total and main SHAP values indicates potential effect modification, suggesting the feature’s impact depends on other variable values. Interaction SHAP values quantified joint feature-pair contributions, revealing synergistic (positive) or antagonistic (negative) effects between variables.

Several sensitivity analyses based on the traditional Cox model were conducted to assess the robustness of results. Survival curves characterized group differences using Kaplan–Meier methods for unadjusted estimates and direct adjustment methods for multivariate-adjusted estimates (14, 15). Hazard ratios (HRs) from Cox proportional hazards models quantified relative risks, while absolute survival probabilities at 10 years provided clinically interpretable measures. The adjusted survival curves were generated using the direct adjustment method implemented through the adjusted Curves R package, which marginalizes over confounding variables by standardizing multivariable Cox model predictions to the study population’s covariate distribution.

Restricted cubic spline (RCS) Cox models (16) with three knots (10th, 50th, 90th percentiles of age distribution) examined the HR of surgery across the age continuum. In addition, incidence rates (1,000 person-years death rate) for surgical approach across age were estimated using a three-knot RCS Poisson model (17). The knots for the RCS models were strategically placed at the 10th, 50th, and 90th percentiles of the age distribution. The nonlinearity of the association was assessed using a Wald test. Nonlinear patterns in RCS analysis indicate interactions between variables, with curve inflection points representing critical thresholds that fundamentally alter these relationships.

Interaction effects between surgical approach and age were examined on multiplicative and additive scales (18, 19). The multiplicative interaction assesses whether the combined effect differs from the product of individual effects (calculated as HR11/(HR10 × HR01), where HR11 represents both exposures present, and HR10 and HR01 represent single exposures). The additive interaction, measured by the relative excess risk due to interaction (RERI = HR11 – HR10 – HR01 + 1), quantifies whether the combined effect exceeds the sum of individual effects. Notably, RCS analyses served for exploratory hypothesis generation and threshold identification, while dichotomized Cox models provided formal statistical inference for interaction effects.

Baseline characteristics were compared between surgery groups using *χ*^2^ tests for categorical variables and Mann–Whitney U tests for continuous variables. Data were presented as frequencies (percentages) for categorical variables and medians (interquartile ranges) for continuous variables. All analyses utilized R version 4.4.3. The ranger package implemented RSF models, while the treeshap package computed SHAP values for survival outcomes. Cox models and RCSs employed survival and rms packages, respectively. Statistical significance was defined using a two-sided *P*-value threshold of <0.05. Results were considered significant at the following levels: ***P < 0.001, **P < 0.01, and *P < 0.05.

## Results

### Patient characteristics

A total of 6,920 patients with T3b disease who underwent surgical resection met the inclusion and exclusion criteria from the SEER database (Supplementary Fig. 1). As shown in Table 1, total thyroidectomy was performed in 6,638 (95.9%) patients, while 282 (4.1%) underwent lobectomy. Patients undergoing lobectomy were significantly older (median 52 vs 49 years, *P* = 0.003) and more likely to have node-negative disease (81.6 vs 45.9%, *P* < 0.001) compared to those receiving total thyroidectomy. Tumor size did not differ significantly between groups (median 20 vs 21 mm, *P* = 0.074). Multifocality was less common in the lobectomy group (25.5 vs 42.0%, *P* < 0.001), and RAI therapy was administered less frequently (41.1 vs 75.5%, *P* < 0.001).

**Table 1:** Characteristics between patients according to surgical approach in T3b differentiated thyroid cancer.

Variable	Overall	Lobectomy	Total thyroidectomy	*P*-value^†^
	*n* = 6,920*	*n* = 282*	*n* = 6,638*	
Age (years)	49.00 (38.00, 60.00)	52.00 (40.00, 63.00)	49.00 (38.00, 60.00)	0.003^‡^
Age category (years)				0.027
(18, 45)	2,683 (38.8%)	90 (31.9%)	2,593 (39.1%)	
(45, 55)	1,620 (23.4%)	66 (23.4%)	1,554 (23.4%)	
(55, 90+)	2,617 (37.8%)	126 (44.7%)	2,491 (37.5%)	
Sex				0.220
Female	5,082 (73.4%)	216 (76.6%)	4,866 (73.3%)	
Male	1,838 (26.6%)	66 (23.4%)	1,772 (26.7%)	
Race				0.901
White	5,520 (79.8%)	222 (78.7%)	5,298 (79.8%)	
Black	263 (3.8%)	11 (3.9%)	252 (3.8%)	
Other/unknown	1,137 (16.4%)	49 (17.4%)	1,088 (16.4%)	
Marital status				0.400
Married	4,317 (62.4%)	171 (60.6%)	4,146 (62.5%)	
Single	1,512 (21.8%)	57 (20.2%)	1,455 (21.9%)	
Divorced/widowed/separated	859 (12.4%)	44 (15.6%)	815 (12.3%)	
Unknown	232 (3.4%)	10 (3.5%)	222 (3.3%)	
Household income				0.396
Low	1,249 (18.0%)	59 (20.9%)	1,190 (17.9%)	
Middle	3,143 (45.4%)	127 (45.0%)	3,016 (45.4%)	
High	2,528 (36.5%)	96 (34.0%)	2,432 (36.6%)	
County type				0.110
Urban	6,381 (92.2%)	253 (89.7%)	6,128 (92.3%)	
Rural	539 (7.8%)	29 (10.3%)	510 (7.7%)	
Grade				0.144
Well differentiated; grade I	824 (11.9%)	28 (9.9%)	796 (12.0%)	
Moderately differentiated; grade II	315 (4.6%)	15 (5.3%)	300 (4.5%)	
Poorly differentiated; grade III	98 (1.4%)	8 (2.8%)	90 (1.4%)	
Unknown	5,683 (82.1%)	231 (81.9%)	5,452 (82.1%)	
Tumor size (mm)	21.00 (14.00, 35.00)	20.00 (10.00, 40.00)	21.00 (14.00, 35.00)	0.074
Tumor size category (mm)				<0.001^‡^
(0, 20)	3,349 (48.4%)	148 (52.5%)	3,201 (48.2%)	
(20, 40)	2,403 (34.7%)	70 (24.8%)	2,333 (35.1%)	
(40, 90+)	1,168 (16.9%)	64 (22.7%)	1,104 (16.6%)	
Multifocality				<0.001^‡^
No	2,317 (33.5%)	135 (47.9%)	2,182 (32.9%)	
Yes	2,858 (41.3%)	72 (25.5%)	2,786 (42.0%)	
Unknown	1,745 (25.2%)	75 (26.6%)	1,670 (25.2%)	
N stage				<0.001^‡^
N0	3,274 (47.3%)	230 (81.6%)	3,044 (45.9%)	
N1a	2,251 (32.5%)	37 (13.1%)	2,214 (33.4%)	
N1b	1,395 (20.2%)	15 (5.3%)	1,380 (20.8%)	
Radiotherapy	5,126 (74.1%)	116 (41.1%)	5,010 (75.5%)	<0.001^‡^
Follow-up (months)	117.00 (52.00, 162.00)	115.00 (50.00, 167.00)	117.00 (52.00, 162.00)	0.882
Cause of deaths				0.018
Alive	6,246 (90.3%)	247 (87.6%)	5,999 (90.4%)	
Thyroid cancer	204 (2.9%)	5 (1.8%)	199 (3.0%)	
Other causes	470 (6.8%)	30 (10.6%)	440 (6.6%)	

* Median (Q1, Q3); *n* (%).

^†^ Wilcoxon rank sum test; Pearson’s Chi-squared test; Fisher’s exact test.

^‡^ *P* < 0.05.

### Effect of surgical approach

**Figure 1:**
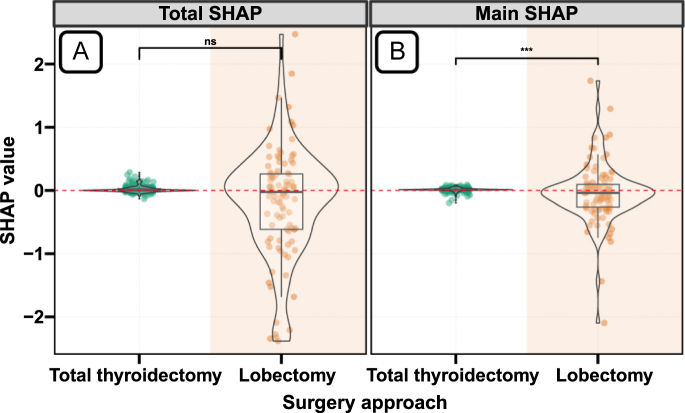
Total and main SHAP contributions for surgery groups using an RSF model. (A) Total SHAP contributions for surgery groups; (B) main SHAP contributions for surgery groups. Mann–Whitney U tests were applied for group comparisons. A full-color version of this figure is available at https://doi.org/10.1530/ERC-25-0417.

The RSF model revealed divergent patterns between total and main SHAP contributions for surgical approach (Fig. 1). Total SHAP values showed no significant difference between lobectomy and total thyroidectomy groups, indicating minimal overall impact of surgical extent on survival when considering all possible variable combinations. However, the main SHAP values, which isolate the direct effect of surgery independent of other variables, demonstrated significant differences between surgical approaches, Traditional Cox regression analyses corroborated the absence of a uniform surgical effect, with no significant survival difference observed between lobectomy and total thyroidectomy in both unadjusted (HR 1.28, 95% CI: 0.91–1.80, *P* = 0.16) and multivariable-adjusted models (HR 0.96, 95% CI: 0.68–1.37, *P* = 0.84) (Supplementary Fig. 2). These findings indicate that the effect of surgical approach is modified by other variables, necessitating further exploration of interaction effects. The notably dispersed distribution of SHAP values in the lobectomy group compared to the relatively concentrated distribution in the total thyroidectomy group further supports this interpretation.

### SHAP interaction analysis

**Figure 2:**
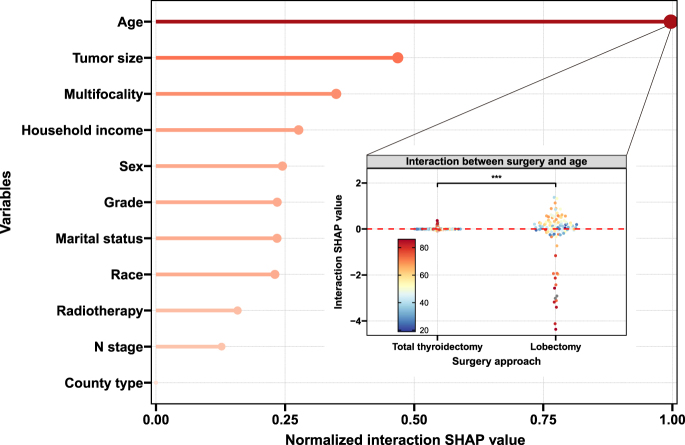
Interaction SHAP contributions between surgery and clinical variables using an RSF model. Mann–Whitney U tests were applied for group comparisons. A full-color version of this figure is available at https://doi.org/10.1530/ERC-25-0417.

The SHAP interaction analysis identified age as the most significant modifier of surgical treatment effect among all clinical variables examined (Fig. 2). The normalized interaction SHAP values demonstrated that the age–surgery interaction contributed more to survival prediction than any other variable combination, including tumor size, nodal status, or multifocality. Critically, this interaction effect was asymmetric between surgical approaches. For patients undergoing lobectomy, interaction SHAP values became progressively more negative with increasing age. In contrast, patients receiving total thyroidectomy showed minimal age-related interaction effects, with values remaining close to zero across the age spectrum. This pattern demonstrates a strong negative interaction between age and surgical approach: the survival benefit of total thyroidectomy over lobectomy is most pronounced in younger patients and progressively diminishes with advancing age until survival differences become negligible.

### Effect of surgical approach across age

**Figure 3:**
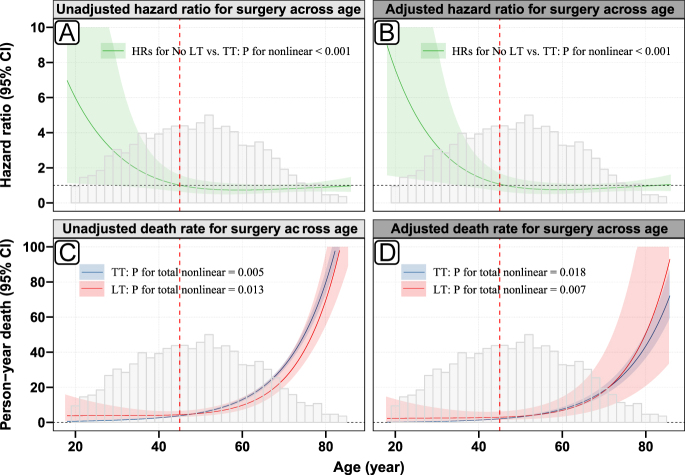
Relative and absolute effect of surgery across age based on RCSs. (A) Unadjusted HR for surgery across age; (B) adjusted HR for surgery across age; (C) unadjusted death rate for surgery across age; (D) adjusted death rate for surgery across age; HR, hazard ratio; LT, lobectomy; TT, total thyroidectomy; the adjusted curves derived from the multivariable Cox model adjusted for sex, race, marital status, household income, county type, histological grade, tumor size, multifocality, N stage, and radiotherapy. A full-color version of this figure is available at https://doi.org/10.1530/ERC-25-0417.

RCS analysis confirmed the age-dependent relationship between surgical approach and survival outcomes (Fig. 3). The HRs for lobectomy versus total thyroidectomy demonstrated significant non-linearity across the age continuum (*P* for non-linearity <0.001). In younger patients, lobectomy was associated with substantially elevated HRs compared to total thyroidectomy. The adjusted HR peaked at approximately 8.0 (95% CI: 2.1–30.5) at age 20, progressively declining with advancing age around 45, where the HR approached 1.0 (95% CI: 0.6–1.7), indicating no significant survival difference between surgical approaches at this threshold. The absolute effect measures (Fig. 3C and D) corroborated these findings through person-years death rates. Specifically, younger patients undergoing lobectomy showed markedly higher mortality rates compared to those receiving total thyroidectomy. The nonlinear pattern provides strong visual evidence of age-dependent treatment heterogeneity and suggests that 45 years represents a critical inflection point.

### Interaction between surgical approach and age

**Figure 4:**
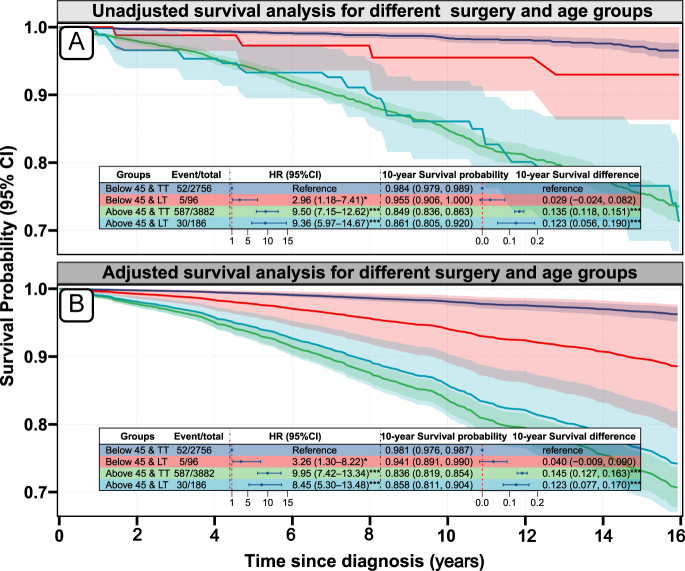
Survival curves for different age and surgery groups based on the Cox model. (A) Unadjusted survival analysis for different surgery and age groups; (B) adjusted survival analysis for different surgery and age groups. HR, hazard ratio; LT, lobectomy; TT, total thyroidectomy; the adjusted curves derived from the multivariable Cox model adjusted for sex, race, marital status, household income, county type, histological grade, tumor size, multifocality, N stage, and radiotherapy. A full-color version of this figure is available at https://doi.org/10.1530/ERC-25-0417.

Survival analysis revealed markedly different effects of surgical approach across age groups (Fig. 4). Among patients younger than 45 years, total thyroidectomy was associated with significantly superior survival compared with lobectomy: 10-year survival probabilities were 98.4% (95% CI: 97.9–98.9) versus 95.5% (95% CI: 90.6–100.0), respectively. This survival disadvantage translated to an HR of 2.96 (95% CI: 1.18–7.41, *P* = 0.021) in unadjusted analysis and 3.26 (95% CI: 1.30–8.22, *P* = 0.012) after multivariable adjustment. Conversely, in patients aged 45 years and older, surgical approach showed no impact on survival. HRs comparing lobectomy to total thyroidectomy were 0.99 (95% CI: 0.68–1.42, *P* = 0.94) in unadjusted and 0.85 (95% CI: 0.58–1.24, *P* = 0.40) in adjusted analyses (Table 2), with virtually overlapped 10-year survival curves between surgical groups.

**Table 2:** Interaction analysis between surgery and age based on Cox model.

	Unadjusted Cox model			Adjusted Cox model		
	Surgery: total thyroidectomy	Surgery: lobectomy	Effect of surgery within the strata of age	Surgery: total thyroidectomy	Surgery: lobectomy	Effect of surgery within the strata of age
	HR (95%CI)	HR (95%CI)	HR (95%CI)	HR (95%CI)	HR (95%CI)	HR (95%CI)
Age: below 45	1.00 (Reference)	2.96 (1.18, 7.41)^‡^	2.96 (1.18, 7.41)^‡^	1.00 (Reference)	3.26 (1.30, 8.22)^‡^	3.26 (1.30, 8.22)^‡^
Age: above 45	9.50 (7.15, 12.62)*	9.36 (5.97, 14.67)*	0.99 (0.68, 1.42)	9.95 (7.42, 13.34)*	8.45 (5.30, 13.48)*	0.85 (0.58, 1.24)
Multiplicative scale	0.33 (0.12, 0.89)^‡^			0.26 (0.10, 0.70)^†^		
RERI	−2.10 (−6.49, 2.29)			−3.76 (−8.23, 0.71)		

Note: multivariable Cox model adjusted for sex, race, marital status, household income, county type, grade, tumor size, multifocality, N stage, and radiotherapy.

Abbreviations: RERI, relative excess risk due to interaction.

* *P* < 0.001.

^†^ *P* < 0.01.

^‡^ *P* < 0.05.

The formal test for interaction confirmed age as a significant effect modifier. The multiplicative interaction term was 0.33 (95% CI: 0.12–0.89, *P* = 0.027) in unadjusted analysis, strengthening to 0.26 (95% CI: 0.10–0.70, *P* = 0.008) after adjustment for confounders. This reduction in the HR between age strata indicates that the survival benefit of total thyroidectomy is restricted to younger patients, while surgical extent becomes clinically irrelevant in older patients with T3b disease. These findings establish age 45 as a critical threshold for personalizing surgical management in T3b DTC.

## Discussion

This population-based analysis of 6,920 patients with T3b DTC demonstrates that surgical approach (lobectomy versus total thyroidectomy) shows no OS benefit when analyzed in aggregate, as confirmed by both RSF total SHAP values and traditional Cox regression (adjusted HR 0.96, 95% CI: 0.68–1.37, *P* = 0.84). However, the divergence between total SHAP (no difference) and main SHAP (significant difference) values indicates that surgery’s impact is entirely dependent on effect modification by other variables, particularly age. Subsequent stratified analyses demonstrated that among patients younger than 45 years (*n* = 2,683; 38.8%), total thyroidectomy was associated with significantly better survival (adjusted HR 3.26, 95% CI: 1.30–8.22, *P* = 0.012), whereas no effect was observed in patients aged 45 years and older (*n* = 4,237; 61.2%) (adjusted HR 0.85, 95% CI: 0.58–1.24, *P* = 0.40). The significant negative multiplicative interaction (0.26, 95% CI: 0.10–0.70, *P* = 0.008) quantifies how age fundamentally alters the relationship between surgical extent and survival. These findings challenge current guidelines recommending universal total thyroidectomy for T3b disease and highlight the critical importance of examining treatment effect heterogeneity rather than relying solely on aggregate analyses.

The divergent surgical outcomes across age groups align with fundamental differences in thyroid cancer biology throughout the lifespan. In our study, total thyroidectomy was associated with a 10-year survival of 98.4% in patients under 45 years, which was numerically higher than the 95.5% rate associated with lobectomy (although the difference was not statistically significant). This survival advantage likely reflects multiple mechanisms: the high prevalence of multifocal disease that may harbor additional foci of strap muscle invasion, the facilitation of effective RAI therapy requiring complete thyroid ablation, and the elimination of potential sources for dedifferentiation over the longer expected lifespan of younger patients. Recent molecular analyses have demonstrated that younger patients with T3b disease maintain higher sodium-iodide symporter expression and greater RAI avidity, potentially explaining why total thyroidectomy-facilitated RAI therapy provides meaningful benefit in this population (20). Younger patients face longer life expectancy during which residual thyroid tissue could undergo dedifferentiation or accumulate additional oncogenic mutations, representing a long-term risk eliminated by complete thyroidectomy (13). BRAF V600E mutations, while equally prevalent across age groups in T3b disease, may have different functional consequences depending on patient age and concurrent molecular alterations (21, 22). Furthermore, the tumor microenvironment differs substantially between younger and older patients, with age-related changes in immune surveillance, angiogenesis, and stromal composition potentially influencing both local invasion patterns and treatment responsiveness (23, 24).

Conversely, the absence of a survival difference between surgical approaches in patients aged 45 years and older suggests that factors beyond surgical extent dominate prognosis in this population. Older patients may harbor more aggressive tumor biology with reduced iodine avidity, limiting the benefits of total thyroidectomy-facilitated RAI therapy (25). In addition, competing mortality risks from cardiovascular disease and other comorbidities become increasingly dominant with advancing age. Our data showed that while thyroid cancer-specific deaths comprised only 2.9% of all deaths, other-cause mortality accounted for 6.8%, potentially masking any modest survival advantage from more extensive surgery. These mechanistic considerations align with our empirical findings and suggest that age-stratified treatment recommendations reflect fundamental biological heterogeneity.

The identification of age 45 as the critical threshold for surgical decision-making carries particular significance given ongoing debates about age cutoffs in thyroid cancer staging. While the AJCC 8th edition shifted from age 45–55 years for staging purposes, our data suggest that age 45 remains the more appropriate inflection point for treatment decisions in T3b disease. The SHAP interaction analysis revealed that the negative interaction between age and lobectomy began precisely around age 45, with progressively negative values thereafter. This threshold effect was consistent across multiple analytical approaches – RSF models, traditional Cox regression, and RCS analysis – strengthening confidence in this age-based treatment boundary.

Our findings reconcile apparent contradictions in the existing literature regarding T3b surgical management. Previous studies reporting favorable outcomes with lobectomy often included older patients with favorable selection characteristics (26, 27), as evidenced in our cohort where lobectomy patients were significantly older (median 52 versus 49 years) and more likely node-negative (81.6 versus 45.9%). The older average age observed in the lobectomy group may be explained by several factors. First, older patients often have a higher perioperative risk due to medical comorbidities, leading surgeons and patients to favor lobectomy as a less extensive and less morbid surgical option (28). Second, this pattern reflects real-world clinical decision-making, as clinical practice over the study period (2004–2022) increasingly recognized that older patients with differentiated thyroid cancer derive limited oncologic benefit from more aggressive surgery (29). Third, for older patients without high-risk features, the disease tends to exhibit more indolent behavior and a generally favorable prognosis, further encouraging the use of more conservative surgical approaches in this population (30). Importantly, these selection patterns do not undermine the validity of our findings. The age-stratified analyses and multivariable adjustments applied in this study effectively accounted for such baseline imbalances. When we stratified by age, the apparent equivalence between surgical approaches disappeared in younger patients, explaining why studies with younger cohorts tend to favor total thyroidectomy while those with older populations show no difference. The recent study demonstrating that strap muscle invasion lacks prognostic significance without other high-risk features particularly applies to older patients (31), where our data confirm that surgical extent becomes irrelevant to outcomes.

From a clinical practice perspective, our findings support nuanced surgical decision-making that considers patient age as a primary stratifying factor. For patients younger than 45 years with T3b disease, total thyroidectomy should remain the standard approach given the significant survival benefit and acceptable surgical risk profile in this population. The absolute survival difference of 2.9% at 10 years, while modest, represents a meaningful benefit in young patients with decades of life expectancy. Conversely, for patients aged 45 years and older, lobectomy emerges as a reasonable alternative that avoids bilateral surgery risks without compromising oncologic outcomes. The implications for adjuvant therapy planning are equally important. Our observation that 75.5% of total thyroidectomy patients received RAI versus 41.1% of lobectomy patients reflects current practice patterns, but our results suggest this may represent overtreatment in older patients. Given the absence of survival benefit from total thyroidectomy in patients ≥45 years, routine RAI therapy in this population warrants reconsideration, particularly for those without additional high-risk features. This age-stratified approach aligns with broader trends toward risk-adapted management in thyroid cancer while providing specific evidence for the T3b population.

Several methodological strengths distinguish our investigation, including the largest T3b cohort analyzed to date with sufficient outcome events for robust statistical inference, novel application of machine learning approaches to identify complex interactions that traditional methods miss, comprehensive validation through multiple analytical approaches ensuring consistency of findings, and long-term follow-up capturing the natural history of differentiated thyroid cancer. Importantly, despite the larger proportion of older patients (61.2% aged ≥45 years) and substantially more outcome events in this age group (440 versus 234 deaths), we detected no survival difference between surgical approaches, effectively excluding the possibility that our null findings resulted from inadequate statistical power. The consistency between SHAP analysis, Cox regression, and RCSs strengthens confidence in the age–surgery interaction.

Nevertheless, important limitations merit consideration. The retrospective nature of SEER analysis introduces potential selection bias, as surgical decisions likely reflected unmeasured patient and surgeon factors. The relatively small lobectomy cohort in younger patients (*n* = 96) limits the precision of effect estimates in this critical subgroup. Given the greater comorbidity burden among older patients, residual confounding from unmeasured comorbidity-related factors cannot be excluded and warrants further evaluation in well-designed prospective studies. In addition, SEER lacks detailed information on surgical indication, extent of strap muscle involvement, completeness of resection, and postoperative thyroglobulin trends that could refine risk stratification. The absence of molecular marker data, particularly BRAF and TERT promoter mutations, prevents integration of genetic factors that may further modify surgical outcomes. Furthermore, quality-of-life metrics and patient-reported outcomes, increasingly recognized as important endpoints in thyroid cancer, remain unmeasured in population databases. We also acknowledge that the mechanistic explanations for the age-specific differences in surgical impact require further validation through future translational studies incorporating genomic profiling and tumor microenvironment analyses.

## Conclusion

This study demonstrates that the surgical approach shows no significant survival difference in T3b DTC, which is masked by a negative age-dependent interaction. The effect of lobectomy versus total thyroidectomy progressively diminishes with advancing age, with total thyroidectomy benefiting only patients younger than 45 years. These findings challenge universal total thyroidectomy recommendations and support age-stratified surgical decision-making for T3b DTC.

## Supplementary materials



## Declaration of interest

The authors declare that there is no conflict of interest that could be perceived as prejudicing the impartiality of the work reported.

## Funding

This work did not receive any specific grant from any funding agency in the public, commercial, or not-for-profit sector.

## Author contribution statement

Yifan Liu was responsible for conceptualization, data curation, project administration, validation, writing the original draft, and writing review and editing. Zhuo Chen was responsible for conceptualization, methodology, data curation, formal analysis, visualization, project administration, validation, writing the original draft, and writing review and editing. Botao Sun was responsible for data curation and formal analysis. Hui Ouyang was responsible for conceptualization, supervision, writing review and editing, and project administration. All authors approved the final version of the manuscript.

## Data availability

The datasets used and/or analyzed for the present study are available from the corresponding author (Hui Ouyang, 228102132@csu.edu.cn) on reasonable request.

## Ethical approval

Given the publicly available nature of the SEER database, this study did not require review by the Institutional Review Board of Central South University.
