# Supplementary material for: Age-dependent effects of surgical approach in T3b differentiated thyroid carcinoma: a population-based analysis using machine learning
Source: Endocr Relat Cancer. 2025 Dec 17;32(12):e250417. doi: 10.1530/ERC-25-0417 (PMC12910566; doi:10.1530/ERC-25-0417)

Age-Dependent Effects of Surgical Approach in T3b Differentiated Thyroid Carcinoma: A Population-Based Analysis Using Machine Learning

Supplementary Materials

Supplementary Figure 1. Flow diagram of study patients. DTC, differentiated thyroid carcinoma.

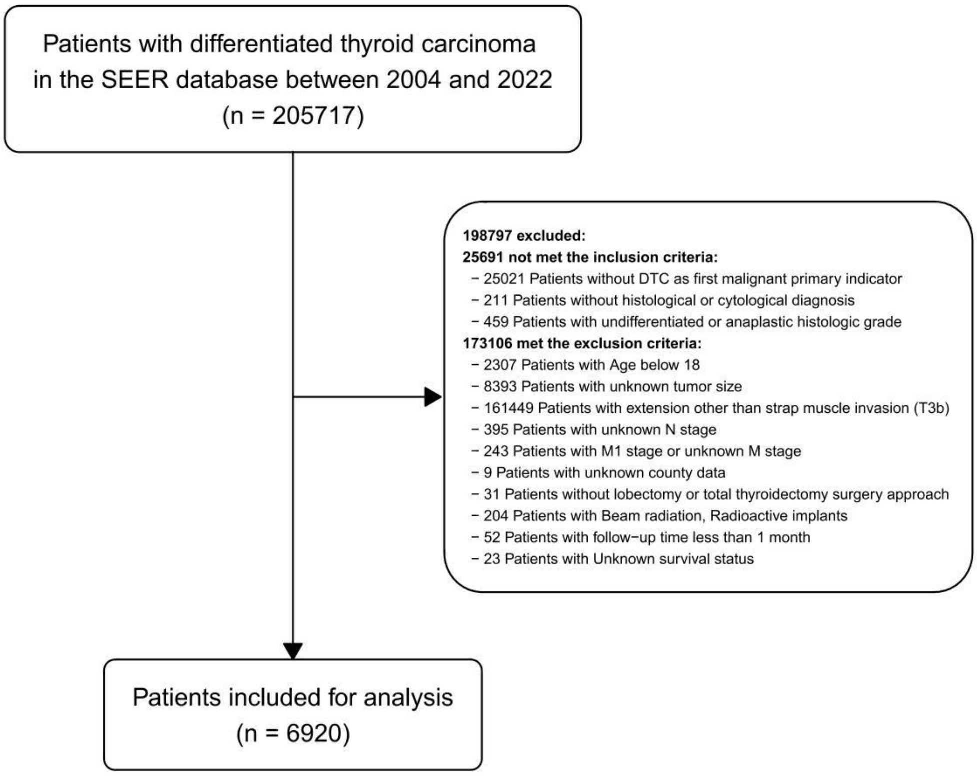

Supplementary Figure 2. Survival curves for surgery groups based on the Cox model. HR, hazard ratio; LT, lobectomy; TT, total thyroidectomy; The adjusted curves derived from multivariable Cox model adjusted for age, sex, race, marital status, household income, county type, histological grade, tumor size, multifocality, N stage, and radiotherapy.

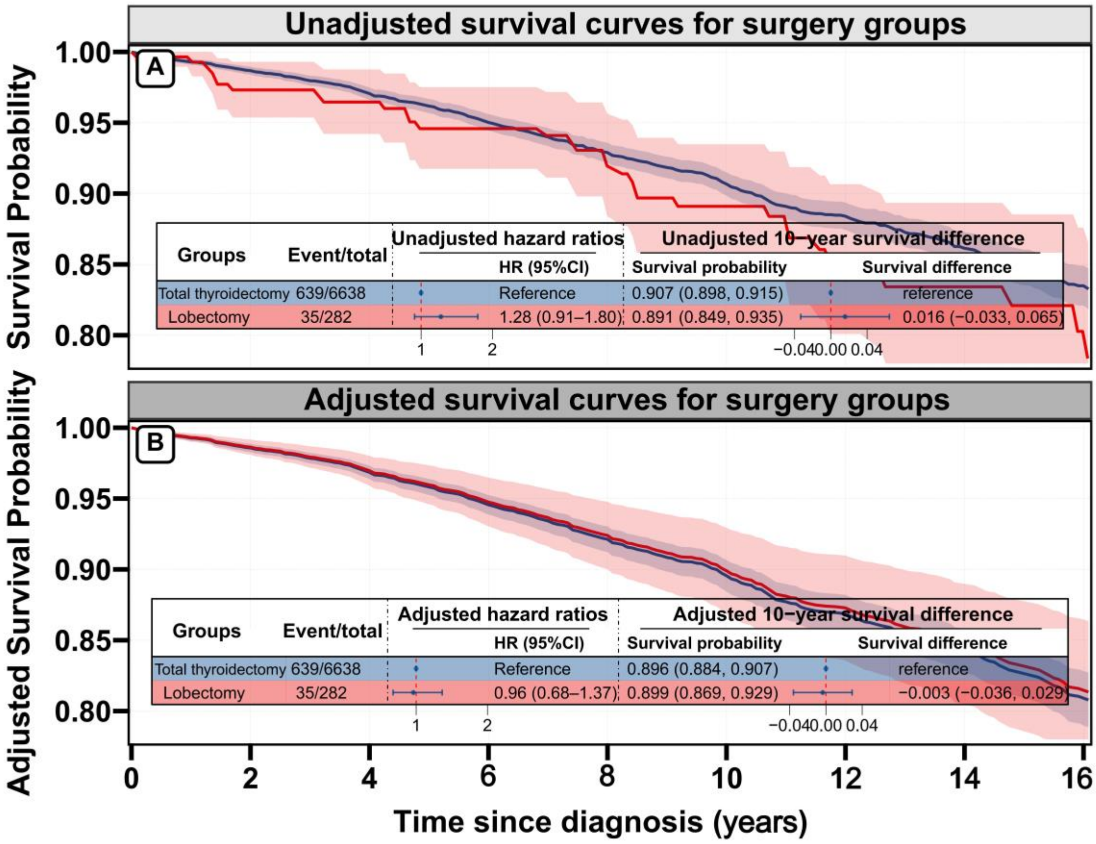

Supplement: Supplementary file 1 [file supplementary_materials.pdf]
